# Supplementary material for: Estimating high-resolution albedo for urban applications
Source: Nat Commun. 2026 Jun 22;17:4815. doi: 10.1038/s41467-026-73436-y (PMC13287749; doi:10.1038/s41467-026-73436-y)
Supplement: Supplementary file 1 — Supplementary Information [file 41467_2026_73436_MOESM1_ESM.pdf]

Estimating high-resolution albedo for urban applications

David Fork[1,†], Elizabeth J. Wesley[2,*], Salil Banerjee[1], Vishal Batchu[1], Aniruddh Chennapragada[1], Kevin Crossan[1], Bryce Cronkite-Ratcliff[1], Ellie Delich[1], Tristan Goulden[3], Mansi Kansal[1], Jonas Kemp[1], Eric Mackres[2], Yael Mayer[1], Becca Milman[1], John C. Platt[1], Shruthi Prabhakara[1], Gautam Prasad[1], Shravya Shetty[1], Charlotte Stanton[1], Wayne Sun[1], Lucy R. Hutyra[1,4]

[1]Google, Mountain View, CA, USA

[2]World Resources Institute, Washington, D.C., USA

[3]National Ecological Observatory Network, Battelle, Boulder, CO, USA

[4]Boston University, Department of Earth & Environment, Boston, MA, USA

[*]Co-Lead Authors

[†]Corresponding Author: David Fork (fork@google.com)

List of Supplementary Items

Supplementary Note 1 3
Supplementary Note 2 4
Supplementary Figure 1 5
Supplementary Figure 2 6
Supplementary Table 2 7
Supplementary References 7

Supplementary Note 1: National Ecological Information Network

The National Ecological Observatory Network (NEON) program, funded solely by the National Science Foundation (NSF), offers free, open, and high-quality data for characterizing ecological processes across 81 field sites in the continental U.S., Puerto Rico, and Hawaii. NEON utilizes an Aerial Observation Platform (AOP), which includes a NextGen NASA Jet Propulsion Lab Airborne Visible InfraRed Imaging Spectrometer (JPL AVIRIS) mounted on a Twin Otter aircraft. This system employs a visible-to-shortwave infrared (VSWIR) pushbroom sensor and acquires data at a 1-m pixel resolution. NEON provides albedo data, defined as the "total amount of solar radiation in the 0.4 to 2.5 micron band reflected by the Earth surface into an upward hemisphere divided by the total amount incident from this hemisphere". This is considered the "best practically achievable albedo measurement" and represents the wavelength-integrated surface reflectance, weighted with the global flux on the ground. NEON albedo data are processed using ATCOR and are provided by flight line. The estimated uncertainty of NEON albedo is ± 0.08 (Hulslander and Thibault).

Supplementary Table 1: Solar geometry of NEON flightlines acquired on May 8, 2024.

Flightline	Solar Azimuth	Solar Elevation
L001	104.09	42.65
L002	105.47	43.89
L003	106.85	45.09
L004	108.38	46.36
L005	110.10	47.72
L006	111.67	48.91
L007	113.29	50.06
L008	115.05	51.28
L009	116.79	52.39
L010	118.71	53.57
L011	120.60	54.64
L012	122.70	55.78
L013	124.69	56.75
L014	126.30	57.58
L016	131.52	59.70
L017	133.88	60.56
L018	136.57	61.45

Supplementary Note 2: Artifacts

There are many algorithms that one might employ to radiometrically calibrate a high-resolution image with a low-resolution image. The most sophisticated methods would account for scene features and artifacts including:

- **imperfect registration:** Often referred to as orthorectification, prior to its use as geospatial imagery, raw overhead imagery is processed to associate each pixel with (longitude, latitude) coordinates based on the camera position and angles and the image height. The process is never perfect; and misregistration can vary from decimeters to tens of meters depending on the data product.
- **non-nadir, oblique views of the scene:** Oblique views (large view zenith angle) displace the tops of buildings by an amount equal to h tan() relative to their bases and for tall buildings in particular, will misregister image pixels with building outlines. In our Boulder study we employed a digital surface model to displace the rooftops onto the building foundation and we masked out the portions of the image that were obscured by the relatively taller objects in both the low- and high-resolution images.
- **varied illumination:** it is rare for two imagers to fly over the same scene at exactly the same time. Due to their complex surfaces, urban features that are imaged at different times will produce a different albedo. This effect occurs not only because some surfaces have a complicated bidirectional reflectance distribution function (BRDF) but also simply because the shadows cast by three dimensional objects will also vary. The Sentinel-2 satellite has a sun synchronous orbit whose nadir view occurs at approximately 10:30 AM local solar time. Certain other sun-synchronous satellites have the same approximate local solar flyover time, including the Pléiades Neo satellite product used in this study. When flyover times agree, this will tend to minimize the differences in shadows between images, but it will not eliminate the effect of shadows on the measurement of albedo.
- **varied and unknown surface shape:** a common assumption built into remote sensing albedo models is that the surface being measured is either flat or lies flat against the terrain. This is untrue for buildings, especially buildings with steep roofs. Where we have roof pitch and azimuth data, we apply the surface albedo correction described in the Methods section.
- **change over time:** no urban scene is static. On a minute-by-minute basis, vehicles come and go; this renders features like roads and parking lots variable over even short intervals. Generative AI may be useful for removing such variable artifacts, but only at resolutions where the objects can be detected. On longer time intervals, human activity, especially construction, varies the built environment and its albedo as does soiling and natural alteration to vegetation, especially wet/dry seasons. This is one reason why this paper has focused on buildings even though cool pavements have a significant potential to cool cities in addition to cool roofs.
- **non-Lambertian surfaces:** many surfaces within a scene tend to be smooth or shiny including metal roofs. As a result, these features can appear to be very dark when the direct solar irradiance reflects out of the imager and appear anomalously bright in rare events where specular reflection hits the imager. There is currently no method employed here to attempt to identify and correct for surfaces that are non-Lambertian.

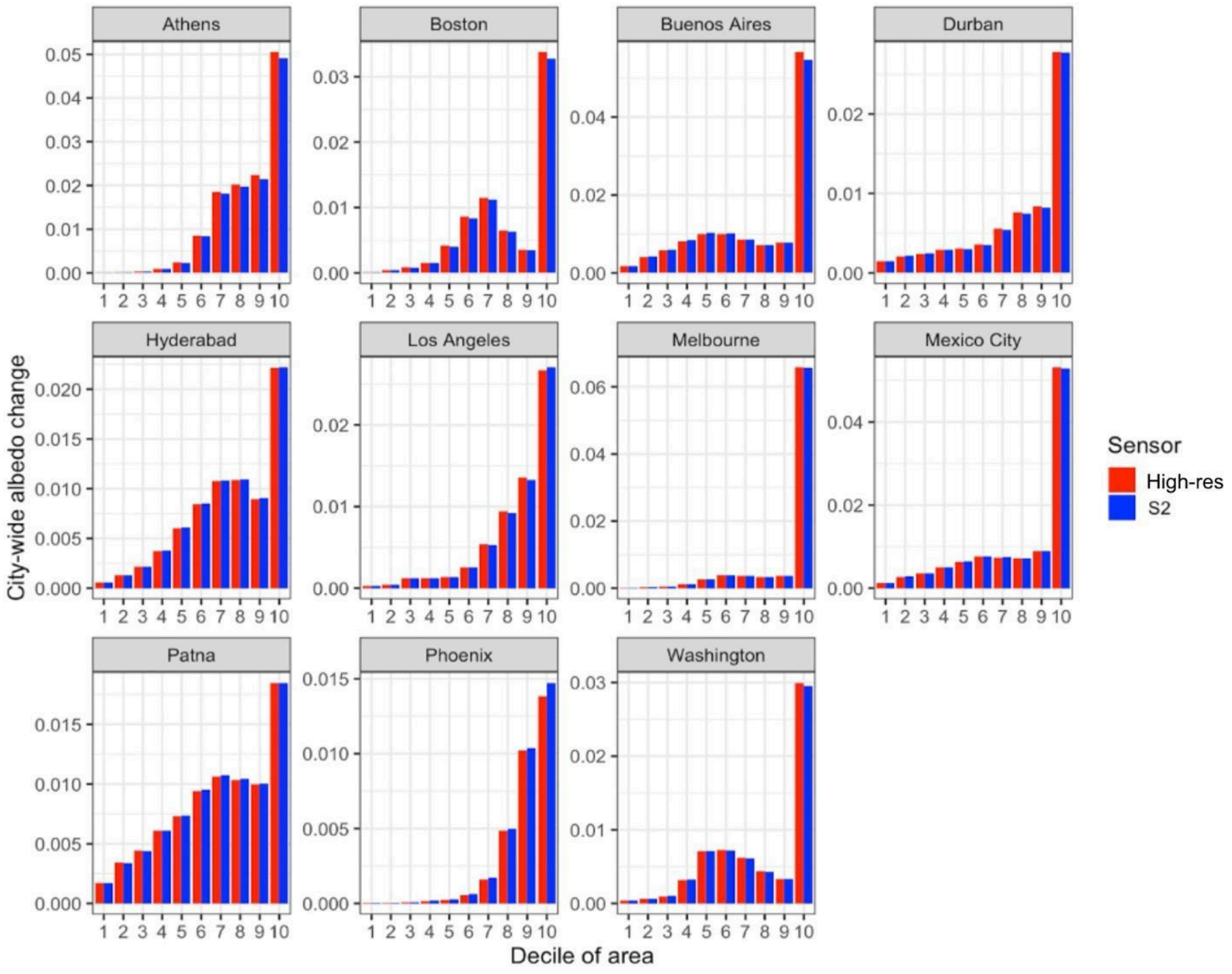

Supplementary Figure 1: City-wide albedo increase resulting from implementing cool roofs (albedo 0.55) on buildings within each decile of building footprint area. Prioritizing buildings in the top 10% of building size could be an effective heat mitigation policy. High-resolution albedo (high-res) shown in red, Sentinel-2 albedo (S2) shown in blue.

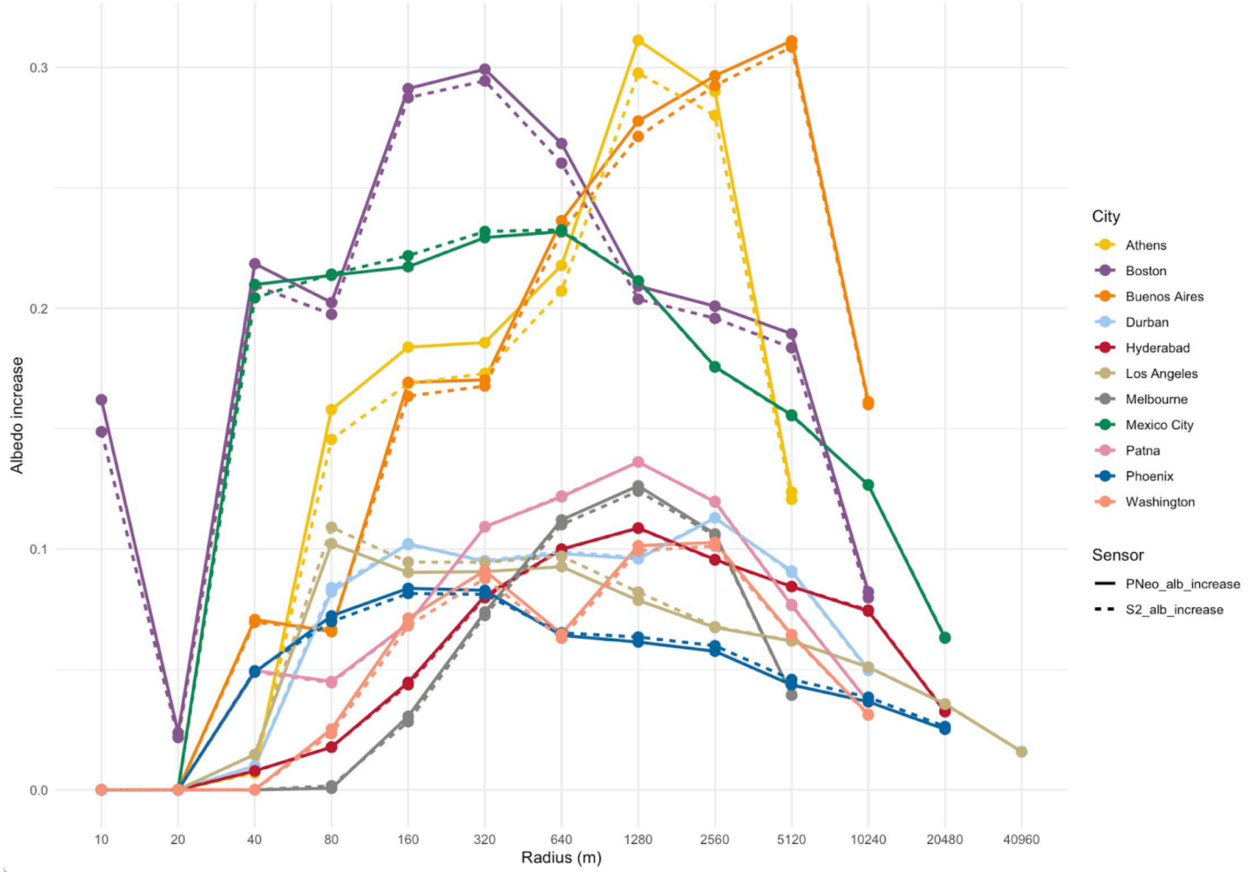

Supplementary Figure 2: Albedo increase resulting from cool roof implementation (albedo of 0.55) calculated for a series of circular buffers with a radius of 10 * 2^x m^2 of the geographic center of the city. The high-resolution albedo (Pneo) and Sentinel-2 (S2) produce similar results regardless of the spatial scale for most cities. Athens is the exception, with Sentinel-2 underestimating the albedo change for radii from 80 to 2560 m.

Supplementary Table 2: Goodness of fit metrics comparing the convolution calibration, calibration transfer, and the Sentinel-2 albedo estimates to the May 8, 2024 NEON ground truth data. The albedos are calibrated from a single, cloud-free Sentinel-2 image from April 30, 2024.

High-res Date	N	Albedo Type	R^2 (Convolution)	R^2 (Transfer)	R^2 (Sentinel-2)	RMSE (Convolution)	RMSE (Transfer)	RMSE (Sentinel-2)	Bias (Convolution)	Bias (Transfer)	Bias (Sentinel-2)
May 5, 2023	11202	Image	0.818	0.837	0.678	0.049	0.056	0.065	0.007	0.032	0.017
May 5, 2023	11202	Surface	0.813	0.828	0.654	0.050	0.059	0.067	0.010	0.035	0.020
May 8, 2024	440	Image	0.918	0.927	0.767	0.046	0.048	0.080	-0.004	0.018	-0.007
May 8, 2024	440	Surface	0.920	0.927	0.762	0.046	0.049	0.081	-0.003	0.019	-0.006
May 16, 2024	15396	Image	0.891	0.900	0.674	0.034	0.038	0.063	0.006	0.015	0.019
May 16, 2024	15396	Surface	0.892	0.898	0.637	0.034	0.039	0.066	0.009	0.018	0.023

Supplementary References

Hulslander, D., & Thibault, K. (n.d.). *NEON ALGORITHMTHEORETICAL BASIS DOCUMENT (ATBD): IMAGING SPECTROMETER ALBEDO*.
