# Supplementary material for: Estimating high-resolution albedo for urban applications
Source: Nat Commun. 2026 Jun 22;17:4815. doi: 10.1038/s41467-026-73436-y (PMC13287749; doi:10.1038/s41467-026-73436-y)
Supplement: Supplementary file 3 — Transparent Peer Review file [file 41467_2026_73436_MOESM3_ESM.pdf]

nature portfolio

Peer Review File

Estimating high-resolution albedo for urban applications

Corresponding Author: Dr David Fork

This file contains all reviewer reports in order by version, followed by all author rebuttals in order by version.

Version 0:

Reviewer comments:

Reviewer #2

(Remarks to the Author)
This paper presents a methodology for enhancing the spatial resolution of albedo estimates for urban applications by combining Sentinel-2 multispectral data with high-resolution satellite imagery. The study demonstrates the method's practical utility by analyzing cool roof implementation scenarios across 12 global cities. This research direction is interesting and significant. However, no particularly significant conclusions were reached. And the reviewer believes that the methodology presented in the paper requires further clarification due to issues such as limited city-scale validation, data time mismatch, and overly simplistic methodology that impact the reliability and universality of the results. The detailed comments are as follows:
1. Global cities exhibit diverse atmospheric conditions, surface characteristics, and urban morphologies. However, this study only conducted accuracy validation in a limited area of Boulder, Colorado, where local atmospheric clarity, roof material distributions, and urban layouts may not represent the full spectrum of global city environments. Since no validation was performed in diverse environments, it is unclear whether the calibration parameters derived in Boulder will be applicable to different urban scenarios or different atmospheric scattering conditions.
2. In the method validation, the high-precision validation subset contains only 440 roof segments, which is insufficient to support robust methodology validation, particularly in areas with diverse roofing materials and geometric variations. A more robust validation would entail stratified sampling across material classes, roof slopes, and building heights, ideally exceeding several thousand segments and spanning multiple seasons to capture phenological and maintenance-driven albedo shifts.
3. Due to the difficulty of acquiring cloud-free Sentinel-2 imagery, the authors adopted a 100-day temporal median approach. However, the large temporal span inevitably affects the accuracy of albedo estimation due to seasonal changes, vegetation phenology, and other temporal factors.
4. The paper assumes rooftop surfaces are Lambertian, which represents an oversimplification. Real urban roofing materials exhibit different reflectance patterns. By leveraging high-resolution imagery and automated methods, even basic classification of rooftop surfaces into categories such as smooth (e.g., metal), rough (e.g., shingles), and vegetated (e.g., green roofs) could help reduce systematic calibration offsets and improve method accuracy.
5. The roof segmentation result shown in the upper right corner of Figure 1 looks rough and contains some errors. Will these errors be further corrected to avoid affecting the subsequent calculation of the roof surface normal?
6. In dense urban districts, inter-building shadows and occlusions significantly alter received radiance, particularly during low-sun-angle periods. The current workflow applies calibration without adjusting for pixel-level shading and does not consider information such as the height of individual buildings, which may lead to underestimation of roof albedo in shadowed zones and overestimation in adjacent sunlit surfaces. Incorporating data such as solar incidence angle and building footprint and height products would help further improve the accuracy of reflectance estimates.
7. In large-scale applications, computational costs may affect model scalability. The authors should include discussion of method complexity and computational costs in the paper, such as comparing the accuracy and efficiency of convolution calibration versus calibration transfer, which would help in selecting appropriate methods for specific application scenarios.
8. While the study quantifies potential temperature reductions, it stops short of evaluating implementation costs against projected energy savings or health benefits. A cost-benefit matrix linking degrees Celsius of cooling to dollars per square meter of applied cool roofing would empower policymakers to prioritize interventions in budget-constrained scenarios.

(Remarks on code availability)
Seems no code provided.

Reviewer #3

(Remarks to the Author)
The paper presents a way to compute high-resolution urban albedo with two different calibration methods and then compare this new estimate with Sentinel-2 low-resolution data. Increase in roof albedo is a potential geoengineering approach to mitigate climate change effects in cities and, therefore, the treated topic is of certain interest for both scientists and urban planners. The methodology is sound and the results consitent. The reviewer has however some comments to make:

1) The topic has been treated extensively in the literature. What's the crucial novelty of the study? Is the take home message that Sentinel-2 can be used for broad urban analysis, while high-resolution albedo is necessary for actual fine-scale planning? Is the calibration methodology? The reviewer suggests to better clarify the main novelty of the study and also to compare (in the discussion section) the cooling found in this paper with estimates made in literature for consistency.

2) At line 63 is suggested that a Sentinel-2 pixel is 1000 m2, but shouldn't it be 10x10=100 m2?

3) Is there a specific reason for the choice of convolutional calibration versus well-established fusion tools such as HySure?

4) Could you please provide a clear reference of the choice of 0.55 as conservative aged albedo at lines 227-228?

5) At line 350 it is reported Fig.(??), could you please specify which figure this refers to?

6) It is only quickly mentioned both in the introduction and in the methods, but I would better clarify that Sentinel-2 albedo is computed following [18].

7) "The city’s high elevation and generally low aerosol concentrations yield an atmospheric profile with radiative properties distinct from lower-altitude or more polluted locations, potentially influencing the observed reflectance characteristics." (366-368). But if reflectance is considered these effects should have been already accounted for in the atmospheric correction process, so this shouldn't generate significant difference between one city and another beside the intrinsic atmospheric correction model error. Can you please clarify the meaning of this sentence?

(Remarks on code availability)

Version 1:

Reviewer comments:

Reviewer #2

(Remarks to the Author)
The author has addressed most of the reviewers' concerns. While the work has certain limitations, the authors have appropriately acknowledged them and incorporated plans to address these aspects in future research. I recommend the acceptance of this paper after the authors address the following minor issues. However, I must say that the methods used in this work is basic and hardly match advanced ones.
1. The manuscript compares "Convolution Calibration" with a "Calibration Transfer" (Machine Learning) approach and ultimately recommends the convolution method. However, since the ML model used for comparison appears to be a relatively basic implementation, I suggest expanding the discussion to acknowledge other existing advanced fusion methods.
2. There is a minor typesetting issue following Equation (2) (around Line 551). The explanatory text starting with "where D is..." appears to be indented as a new paragraph. This clause should be treated as a continuation of the sentence containing the equation and should be left-aligned.

(Remarks on code availability)

Reviewer #3

(Remarks to the Author)
The reviewer finds that the authors have carefully responded to the question posed by the reviewer. The more concerning was about novelty, but the authors pointed out that this is one of the rare cases were contemporaneous albedo images of both satellite and aircraft can be taken into account at the same moment. The reviewer therefore suggests the paper for publication.

(Remarks on code availability)

Reviewer #4

(Remarks to the Author)
I co-reviewed this manuscript with one of the reviewers who provided the listed reports. This is part of the Nature Communications initiative to facilitate training in peer review and to provide appropriate recognition for Early Career Researchers who co-review manuscripts.

(Remarks on code availability)

Open Access This Peer Review File is licensed under a Creative Commons Attribution 4.0 International License, which permits use, sharing, adaptation, distribution and reproduction in any medium or format, as long as you give appropriate credit to the original author(s) and the source, provide a link to the Creative Commons license, and indicate if changes were made.
In cases where reviewers are anonymous, credit should be given to 'Anonymous Referee' and the source.
The images or other third party material in this Peer Review File are included in the article's Creative Commons license, unless indicated otherwise in a credit line to the material. If material is not included in the article's Creative Commons license and your intended use is not permitted by statutory regulation or exceeds the permitted use, you will need to obtain permission directly from the copyright holder.
To view a copy of this license, visit https://creativecommons.org/licenses/by/4.0/

REVIEWER COMMENTS

Reviewer #2 (Remarks to the Author):

This paper presents a methodology for enhancing the spatial resolution of albedo estimates for urban applications by combining Sentinel-2 multispectral data with high-resolution satellite imagery. The study demonstrates the method's practical utility by analyzing cool roof implementation scenarios across 12 global cities. This research direction is interesting and significant. However, no particularly significant conclusions were reached. And the reviewer believes that the methodology presented in the paper requires further clarification due to issues such as limited city-scale validation, data time mismatch, and overly simplistic methodology that impact the reliability and universality of the results. The detailed comments are as follows:

We appreciate the constructive reviewer comments and have worked to address them throughout the paper (see below). In addition, we have clarified and honed the significant objectives and conclusions of the paper in the abstract (lines 19-21, 22-23), at the end of the introduction (lines 107-110), and in the conclusion (lines 471-477, 499-502).

1. Global cities exhibit diverse atmospheric conditions, surface characteristics, and urban morphologies. However, this study only conducted accuracy validation in a limited area of Boulder, Colorado, where local atmospheric clarity, roof material distributions, and urban layouts may not represent the full spectrum of global city environments. Since no validation was performed in diverse environments, it is unclear whether the calibration parameters derived in Boulder will be applicable to different urban scenarios or different atmospheric scattering conditions.

Validation of urban albedo estimates is difficult and extremely rare within the published literature. In this study, we also took the exceeding rare and difficult step of tasking a satellite to acquire Pleiades Neo imagery to temporally align with the NEON hyperspectral flights May 8, 2024 (lines 125-128). NEON hyperspectral flights typically focus on rural ecological observations; we were able to collaborate with NEON to acquire their calibration flight data from Boulder, CO. While this direct validation data may cover only a single city, it is exceptionally powerful 1m urban data that is non-existent in the literature. Ban-Weiss (2015, https://doi.org/10.1016/j.solener.2014.10.022) made a notable attempt towards this type of validation in 2009 with NAIP imagery for cities in California, but the spectral and

spatial resolution was much more limited in that case.   While we would have ideally liked to have used varied validation data, using this validation data is a major advancement in the literature. Tasking additional commercial satellite acquisitions and hyperspectral flights over other cities was beyond the scope of this study.

The processing workflow in our study starts with the Sen2Cor algorithm applied to Sentinel-2 observations to convert the top-of-atmosphere reflectance to a bottom-of-atmosphere reflectance product by applying atmospheric corrections. The Sen2Cor algorithm is supported by the European Space Agency and has been heavily validated using the global Radiometric Calibration Network (Main-Knorn et al. 2017; 10.1117/12.2278218). To convert the narrow Sentinel-2 bands to a broadband albedo estimate we applied the Bonafoni model (10.1109/LGRS.2020.2967085). We also develop and assess the accuracy of two methods for inferring high-resolution albedo through convolutional calibration and calibration transfer. We have added clarification to the manuscript to clarify these points (line 148).

2. In the method validation, the high-precision validation subset contains only 440 roof segments, which is insufficient to support robust methodology validation, particularly in areas with diverse roofing materials and geometric variations. A more robust validation would entail stratified sampling across material classes, roof slopes, and building heights, ideally exceeding several thousand segments and spanning multiple seasons to capture phenological and maintenance-driven albedo shifts.

We agree with the reviewer that many segments are required for a robust validation. While we begin with a sample of 440 roof segments that intersect the area covered by both the single date Sentinel-2 image and high-resolution image closest in time to the NEON flyover, we expand our analysis to the broader sample of 54,076 roof segments covered by other images. We added text to clarify this point (lines 202, 207). Additionally, while stratifying the samples by materials or heights is outside the scope of this analysis, we do examine variation by roof slope, azimuth, and area (lines 253-260) (fig 3).

3. Due to the difficulty of acquiring cloud-free Sentinel-2 imagery, the authors adopted a 100-day temporal median approach. However, the large temporal span inevitably affects the accuracy of albedo estimation due to seasonal changes, vegetation phenology, and other temporal factors.

We kindly direct the reviewer’s attention to Table 2 where we compare the single-day albedo estimates with the temporal median estimate and find a negligible decrease in

accuracy. This is an important (and lucky) feature of our experiment both because May 8, 2024 was a clear sky day in Boulder for the NEON and Pneo flights and because when Sentinel-2 happened to be flying overhead a week earlier on April 30th, the sky was clear.

"Our goal for this research was to develop a calibration method applicable at almost any time and anywhere on Earth and at any time. This requires a time span that consistently produces stable, representative images of surface reflectance, for which we have determined at 100-day medians to be robust." We have added the results of our sensitivity analysis of the dayspan window for median albedo estimation to the SI of the manuscript (Figure SI-2). We show that the results of the single day and 100 day median estimate were not very different from a clear-sky contemporaneous measurement. Importantly, the median statistic is a proven method to remove artifacts due to snow, clouds, and glint from image collections such as Sentinel 2. Compared to the seasonal variation in albedo, the region mean albedo is not strongly dependent on the length of the temporal span used to compute the albedo median. This indicates that even though there is seasonal variation in shadows and vegetation phenology around the mid-date, how far before and after the mid-day one collects data has only a secondary effect on the measurement. The collection of multiple satellite flyovers and the use of methods such as the median statistic or more sophisticated multi-image fusion method may be the only practical method to scale the generation of calibration imagery to cities all over the world, which is the author's objective.

The main paper narrative has also been modified for greater clarity.

4. The paper assumes rooftop surfaces are Lambertian, which represents an oversimplification. Real urban roofing materials exhibit different reflectance patterns. By leveraging high-resolution imagery and automated methods, even basic classification of rooftop surfaces into categories such as smooth (e.g., metal), rough (e.g., shingles), and vegetated (e.g., green roofs) could help reduce systematic calibration offsets and improve method accuracy.

This is an excellent suggestion, however, even the pansharpened 0.3 meter resolution of the PNeo imagery is not currently sufficient to perform this image segmentation task suggestion. We acknowledge this possibility in a discussion of potential future work, but it would not be possible to treat surfaces as non-Lambertian in this study. To our knowledge, this is the first publication that attempts to convert image albedo, that is the albedo assuming a flat reflective scene, into surface albedo, the tilt corrected value of the albedo consistent with the Lambertian assumption. What the reviewer is suggesting is essentially to compute for each pixel a bidirectional reflectance distribution function

(BRDF) and a digital surface model (DSM); one might call this the "holy grail" of urban surface reflectance and is certainly of great interest to the authors. However, reconstructing the BRDF and DSM is a classic inverse problem for which there is insufficient data. One of the interesting possibilities for future work would be to examine the application of ray tracing methods such as inverse rendering to try to extract a BRDF from each point on the surface. Something similar has been done to some extent using the MODIS satellite constellation, but at a resolution of 500 meters, which is not relevant to urban surface reflectance due to the low resolution.

We have included a "future work" section in the narrative to pique interest that some readers may have in further exploring this possibility (line 444-455).

5. The roof segmentation result shown in the upper right corner of Figure 1 looks rough and contains some errors. Will these errors be further corrected to avoid affecting the subsequent calculation of the roof surface normal?

As the cited paper (Batchu et al. 2025) discusses, the roof segmentation represents the state of the art of Google's AI generated, self-aligned digital surface maps. This is both a technical breakthrough and also an area of constant improvement, so yes, in subsequent releases, the roof segmentations will continue to improve. We refer the reviewer to Google's regularly updated Solar API release notes, which in September 23, 2025 announced its Expanded Medium-quality data coverage. As an interesting side note, the roof segmentations Google first produced were generated solely by expensive, aircraft-based high resolution stereoimagery. The imagery was only available in wealthy portions of the US and Europe where aircraft flyovers were economically justifiable. An objective of Project Sunroof and our efforts here has been to extend roof segmentation globally by using relatively inexpensive satellite imagery and advanced machine learning methods to replace the expensive aircraft stereo imager with a product that needs only a single satellite image to generate roof segmentations adequate for screening rooftops for their solar energy generation potential. This was the subject of a blog post in Dec 2024[1] which featured the addition of 125 million new buildings to the Solar API in places such as Malaysia and India and with the potential to add 1.9 billion additional buildings.

6. In dense urban districts, inter-building shadows and occlusions significantly alter received radiance, particularly during low-sun-angle periods. The current workflow applies calibration without adjusting for pixel-level shading and does not consider information such as the height of individual buildings, which may lead to underestimation of roof albedo in shadowed zones and overestimation in

[1] https://research.google/blog/satellite-powered-estimation-of-global-solar-potential/

adjacent sunlit surfaces. Incorporating data such as solar incidence angle and building footprint and height products would help further improve the accuracy of reflectance estimates.

The reviewer makes good points about shadows, occlusions and building height.

- Shadows: PNeo and Sentinel-2 both have the same approximate flyover time, 10:30 AM local solar time, so to first order, the shadowing in the calibration and target imagery is the same. What we report on in this paper is technically called the blue sky albedo, which is a mixture of the black (direct) and white (diffuse) sky albedos. It is within the capabilities of future efforts (but beyond the scope of this work) to use height maps of the buildings to generate a shadow map of the scene.
- Occlusions: Occlusion (and building lean) are important artifacts that can appear in oblique images, particularly for tall structures. As this paper focused on large samples of buildings, the effects of occlusion and lean have a very small effect on aggregated building statistics because most buildings are not tall enough for there to be a significant effect on the median albedo within the building outline. However, the reviewer is correct that when it comes to very tall buildings, and nearby buildings which may be occluded, the effect is that the inferred rooftop albedo can be incorrect. Such measurements nonetheless represent a tiny fraction of a typical city's building stock. Correcting these tall building artifacts is on our production roadmap. The correction of these artifacts in imagery such as PNeo is just starting to become available in state of the art analyses, https://arxiv.org/abs/2408.14400. Unfortunately, at the time of this study, the methods for these corrections were not available in the full set of cities that the authors studied.

We have added a brief discussion of how shadows and occlusions could be treated in the section on future work.

7. In large-scale applications, computational costs may affect model scalability. The authors should include discussion of method complexity and computational costs in the paper, such as comparing the accuracy and efficiency of convolution calibration versus calibration transfer, which would help in selecting appropriate methods for specific application scenarios.

Following this suggestion, we have revised the discussion (line 214-217) to state "As the results show, two methods, an image processing approach and a machine learning approach, give very similar results even though the methods differ significantly. However, the calibration transfer method had two drawbacks in that it required training a

new model for each high resolution image and for high/ and low albedos, it introduced bias due to regression toward the mean.”

8. While the study quantifies potential temperature reductions, it stops short of evaluating implementation costs against projected energy savings or health benefits. A cost-benefit matrix linking degrees Celsius of cooling to dollars per square meter of applied cool roofing would empower policymakers to prioritize interventions in budget-constrained scenarios.

We very much agree with the reviewer that a cost-benefit analysis of this sort would be possible and we do hope to undertake such work in the future. In Smith et al. (2025; https://doi.org/10.1038/s43247-025-02462-3) we do an analysis similar to this suggestion for a single city and it highlights the importance of local building architecture, bioclimate zones, and an array of other critical considerations. Carrying out that analysis in this paper is beyond the scope of this study which demonstrates a powerful new method for albedo estimation. We have added text to clearly highlight potential applications of this data (line 405).

Reviewer #2 (Remarks on code availability):

Seems no code provided.
The code is provided here (https://codeocean.com/capsule/7640337/tree) and data is provided here (https://doi.org/10.7910/DVN/7T7QDH), we apologize that the reviewer was unable to locate it.

Reviewer #3 (Remarks to the Author):

The paper presents a way to compute high-resolution urban albedo with two different calibration methods and then compare this new estimate with Sentinel-2 low-resolution data. Increase in roof albedo is a potential geoengineering approach to mitigate climate change effects in cities and, therefore, the treated topic is of certain interest for both scientists and urban planners. The methodology is sound and the results consitent. The reviewer has however some comments to make:

1) The topic has been treated extensively in the literature. What's the crucial novelty of the study? Is the take home message that Sentinel-2 can be used for broad urban analysis, while high-resolution albedo is necessary for actual fine-scale planning? Is the calibration methodology? The reviewer suggests to

better clarify the main novelty of the study and also to compare (in the discussion section) the cooling found in this paper with estimates made in literature for consistency.

Thank you for this valuable feedback. We have honed the core objectives and messages of the paper in the abstract, at the end of the introduction (line 107-110), and in the conclusion (lines 471-477, 499-502).

With growing constellations of multispectral imagers and the methods developed and described here, it is now possible to measure calibrated building level albedo for the entire world's urban areas and pursue climate adaptation measures informed by these observations. Critically and uniquely, this study includes large scale validation of overhead roof top satellite radiometry measurements with contemporaneous airborne hyperspectral imagery. Demonstration of urban albedo inference using two sun-synchronous satellites, one for calibration, and one for resolution enhancement. This unique demonstration realizes the potential of global urban albedo coverage at high resolution and high repeat cadence. Our analysis gives researchers confidence in using Sentinel-2 albedo estimates for broadscale urban analysis, but fine-scale (building level) planning requires higher resolution data as that described in our paper that will be made available after the publication of this manuscript.

2) At line 63 is suggested that a Sentinel-2 pixel is 1000 m2, but shouldn't it be 10x10=100 m2?

Thank you for helping us clarify our point. The 1000 m2 area does not refer to the area of a Sentinel-2 pixel. Instead, it represents an approximate minimum roof area required for the surface to be captured by a sufficient number of unmixed pixels, thereby enabling an accurate estimate of rooftop albedo. We have revised the wording (line 76-84) to make this clear.

3) Is there a specific reason for the choice of convolutional calibration versus well-established fusion tools such as HySure?

Thank you for directing us to review the HySure software. We chose to use convolutional calibration because when we first used the ML based calibration transfer method, we observed the model regressing toward the mean and hence underestimating high albedo and over estimating low albedo. Convolutional calibration does not have this problem and it is simple and fast and can process large volumes of data quickly in our production pipeline. After careful reviewing, we believe that HySure is indeed an interesting algorithm because it attempts to solve the inverse problem by

optimizing both the spatial and spectral trends. HySolve also has its own barriers to use for this study which include:

1. It is available only in MatLab, which does not run on our production hardware.
2. Its use of ADMM and convex optimization is significantly more complex than convolutional calibration.

The authors acknowledge the reviewer's comment and will ensure that the HySure algorithm gets cited in the revised manuscript (line 211). A comparison of HySure to other methods would indeed be interesting in subsequent studies.

4) Could you please provide a clear reference of the choice of 0.55 as conservative aged albedo at lines 227-228?

There is indeed variability across cities, states, and national programs for the minimum reflectivity value for a cool roof. The text has been clarified (lines 272-275) and a citation has been added that documents 0.55 as a commonly applied standard.

5) At line 350 it is reported Fig.(??), could you please specify which figure this refers to?

Thank you for flagging this typo. We have corrected the figure reference.

6) It is only quickly mentioned both in the introduction and in the methods, but I would better clarify that Sentinel-2 albedo is computed following [18].

The reviewer is correct in flagging that we are not explicit about our use of the Bonafoni and Sekertekin 2020 methods for calculating albedo from Sentinel-2. We have added text at line 138 to clarify.

7) "The city's high elevation and generally low aerosol concentrations yield an atmospheric profile with radiative properties distinct from lower-altitude or more polluted locations, potentially influencing the observed reflectance characteristics." (366-368). But if reflectance is considered these effects should have been already accounted for in the atmospheric correction process, so this shouldn't generate significant difference between one city and another beside the intrinsic atmospheric correction model error. Can you please clarify the meaning of this sentence?

The reviewer is probably correct that we may have been over-cautious in calling out possible limitations to the study we carried out, and thus may have failed to adequately convey the globally scalable nature of this work. We elected to rewrite the limitations section as follows:

This study was validated exclusively in Boulder, Colorado, which may limit the generalizability of the results. Boulder's built environment includes unique characteristics such as specific roof geometries, materials, and building height restrictions that may not be representative of many other urban areas. In particular, the city's stringent height ordinances result in a lack of the kinds of high-rise structures common in denser metropolitan contexts. Additionally, the building density of our validation area is much lower (approximately 6%) than typical urban areas (approximately 20%), potentially reducing the impacts of shadowing. Additionally, the surface properties of Boulder's natural environment, including coloration and bidirectional reflectance (BRDF), are likely to differ from those in other geographic regions. Ground truth validation in multiple cities representing a diversity of surface characteristics and urban morphologies would contribute greatly to the literature.
